# Dental caries and periodontitis and the risk of myopia in young adults: CHIEF oral health study

**DOI:** 10.1186/s12903-022-02413-w

**Published:** 2022-09-05

**Authors:** Kun-Zhe Tsai, Pang-Yen Liu, Yen-Po Lin, Shu-I. Pao, Ming-Cheng Tai, Jiann-Torng Chen, Gen-Min Lin

**Affiliations:** 1 Department of Stomatology of Periodontology, Mackay Memorial Hospital, Taipei, Taiwan; 2 Department of Medicine, Hualien Armed Forces General Hospital, No. 163, Jiali Rd., Xincheng Township, Hualien, 97144 Taiwan; 3 Departments of Dentistry, Tri-Service General Hospital, National Defense Medical Center, Taipei, Taiwan; 4 Graduate Institute of Dental Science, National Defense Medical Center, Taipei, Taiwan; 5 Department of Internal Medicine, Tri-Service General Hospital, National Defense Medical Center, Taipei, Taiwan; 6 Department of Critical Care Medicine, Taipei Tzu-Chi Hospital, New Taipei City, Taiwan; 7 Department of Ophthalmology and Visual Sciences, National Defense Medical Center, Tri-Service General Hospital, Taipei, Taiwan

**Keywords:** Actively dental caries, Filled teeth, Periodontitis, Myopia, Young adults

## Abstract

**Aim:**

Oral health and ocular diseases may be associated with collagen defects and inflammation status. However, the results from prior studies are conflicting. The aim of this study was to explore the association of dental caries and periodontitis with myopia in young adults.

**Materials and methods:**

A total of 938 military personnel aged 19–39 years receiving both oral and eye examinations from 2018 through 2020 were included in this study in Taiwan. The severity of myopia was graded as no myopia (diopters > − 0.5, N = 459), low myopia (diopters: − 0.5 to -5.9, N = 225) and high myopia (diopters ≤ − 6.0, N = 254). A multiple logistic regression analysis with adjustments for age, body mass index, systolic blood pressure, smoking, alcohol consumption, missing teeth numbers, blood leucocyte counts, triglycerides, high-density lipoprotein, and uric acid were used to determine the associations of actively dental caries, filled teeth and stage II/III periodontitis with myopia.

**Results:**

The presence of any actively dental caries was significantly associated with a higher risk of any myopia (low or high) (odds ratio [OR] and 95% confidence intervals [95% CI] 1.42 [1.04–1.94]), whereas there was no association for filled teeth. Moreover, the association for stage II/III periodontitis was only observed with high myopia (OR: 1.52 [1.07–2.15]) and was not observed with low myopia.

**Conclusions:**

Our findings suggest that only actively dental caries and a higher severity of periodontitis were associated with myopia among young adults, thus highlighting the dental inflammation status in the oral cavity as a potential link to ocular diseases.

**Supplementary Information:**

The online version contains supplementary material available at 10.1186/s12903-022-02413-w.

## Introduction

Dental caries and periodontal diseases affect numerous people worldwide, and treatment costs cause a significant burden on health services [1]. Dental caries can cause pain during eating, speaking difficulties, low self-esteem, tooth loss and the need for surgery [2]. In addition, myopia is also a common ocular disorder. Epidemiological studies have demonstrated that the prevalence of myopia has increased in recent decades and has resulted in a significant global public health concern, which has been most dramatically observed among younger people in East Asia [3]. The incidence of myopia in young adults was observed to be greater in Asian and female individuals and progressed with older ages [4]. Although the exact etiology of myopia remains elusive, it appears to be related to interactions between genetic and environmental factors [5], thus making prevention and treatment of myopia a challenging issue.

Several risk factors, such as low physical activity and body weight, may lead to both dental caries and high myopia [6–9]. In addition, systemic inflammation related to periodontitis [10] and actively dental caries [11] may play a role in the process of various ocular diseases. In the 1970s, Goldstein et al. [12] and Hirsh et al. [13] reported that there was a higher risk of dental caries in myopic children than in nonmyopic children. In contrast, in other studies [14–16], these associations were not found. To date, there are only two studies [17, 18] on the association between periodontitis and incident ocular diseases (specifically regarding glaucoma), and the risk ratio was estimated to be 1.26 [17]. However, the extent and severity of periodontitis could not be determined in this insurance data-based study, and the risk of myopia was not examined. Therefore, the present study aimed to examine the associations of dental caries with and without complete dental treatment and periodontitis with myopia.

## Methods and methods

### Study population

The cardiorespiratory fitness and health in eastern armed forces (CHIEF) [19] oral health study [20–23] was performed at the Hualien Armed Forces General Hospital in Hualien County, Taiwan, from 2018 to 2020. The inclusion criteria were those individuals receiving the oral health examination, and the exclusion criteria were those individuals lacking ocular examinations in the same year and those individuals who had a history of ocular surgery. Participants were further divided into three groups: no myopia, low myopia and high myopia.

### Clinical and demographic measurements

Participants were asked to fast for more than 12 h before the annual health examination in the military population. Measurements of anthropometric parameters for body height and body weight were performed in a standing position. Body mass index (BMI) was calculated as body weight in kilograms divided by the square of body height in meters. The systolic and diastolic blood pressure (BP) values of each subject were measured in a sitting position and at rest for at least 15 min by using the same automated blood pressure monitor machine (Parama-Tech Co Ltd, Fukuoka, Japan). The laboratory data for blood total leucocyte counts, fasting plasma glucose, triglycerides, cholesterols and serum uric acid were measured by using the same auto analyzer (Olympus AU640 auto analyzer [Olympus, Kobe, Japan]).

### Dental caries and periodontal condition measurements

A disposable dental mirror (Atlas, Tehran, Iran) and community periodontal index probe (HU Friedy, Chicago, USA) were utilized to examine the teeth. The numbers of decayed, missing and filled teeth (DMFT) were assessed based on the standards defined by the World Health Organization (WHO) (WHO, 2013). Teeth with damaged surfaces or pits and grooves, cavitated enamel and softened surfaces (as detected by the probe) were considered to be actively dental caries. In addition, any tooth that was dressed with one of the temporary fillings and any tooth that was filled but had caries were also considered to be actively dental caries. Furthermore, teeth with faulty sealants were regarded as being actively dental caries. Filled teeth were defined as healing dental caries which have undergone complete treatment and without recurrence. Impacted teeth and third molars were excluded from this study.

Full mouth periodontal charting was recorded at six sites of each tooth, including probing pocket depth (PPD) and clinical attachment loss. In addition, data on the remaining teeth numbers, tooth mobility, furcation involvement and full mouth radiographic images were collected. The severity and grade of periodontitis were classified based on the 2017 world workshop of the American Academy of Periodontology and European Federation of Periodontology [24]. The procedures of all of the dental examinations were performed by the same dentist (Kun-Zhe Tsai) for the evaluation of periodontitis severity and grade. A follow-up examination and treatment for the oral pathologies of each participant was performed in detail by other dentists in the Outpatient Department within one month, and the interobserver agreement (kappa coefficient) for the verification of the stage of periodontitis was estimated to be 90.6%. A comprehensive oral treatment plan was provided to all of the subjects after surveying their baseline oral health. The CHIEF study was reviewed and approved by the Institutional Review Board of the Mennonite Christian Hospital (No. 16–05-008) in Hualien City, Taiwan, and written informed consent was obtained from all of the participants.

### Ocular status measures

The Auto Kerato-Refractometer (KR8100P, Topcon Corporation, Tokyo, Japan) was used to measure noncycloplegic autorefraction of both eyes in each subject, and a Snellen chart was also used to assess each subject’s best corrected visual acuity. At the enlistment prescreening, extreme myopia (≤ − 10.0 diopters), amblyopia, strabismus, a history of high-risk ocular surgery and pathology affecting the clarity of the ocular media were the exclusion criteria. The severity of myopia was graded as low (− 0.5 to − 5.9 diopters) and high (≤ − 6.0 diopters). Other eyes were graded as no myopia (> − 0.5 diopters). Analyses were solely performed by using the left eye measurement, as the association of refractive errors between the left and right eyes was high (r = 0.96) [7].

### Statistical analysis

The sample size was estimated to be 798 participants based on an assumption of an incidence difference in stage II/III periodontitis of 10%, which provides ≥ 80% power (at *p* = 0.05). The characteristics of the participants are presented as the mean ± standard deviation (SD) for continuous data and numbers (%) for categorical data. Continuous variables were compared by using one-way ANOVA, and categorical variables were compared via the χ^2^ test among the myopic groups. Univariate linear regression analysis was used to determine the β value and their 95% confidence interval (CI) of actively dental caries, missing and filled teeth numbers, probing pocket depth, age, BMI, systolic BP, blood leucocyte counts, serum triglycerides, serum high-density lipoprotein and serum uric acid levels with refractive errors (from more negative values [high myopia] to less negative values or zero [low myopia]). Accordingly, if the direction of the β value was positive, the association of the exposure variable with myopia was less likely, and vice versa. Multivariable linear regression model was constructed by backward stepwise selection method. A multiple logistic regression was utilized to determine the odds ratio (OR) of the presence of actively dental caries, filled teeth and stage II/III periodontitis (using health/stage I periodontitis as the reference group) with any myopia, low myopia and high myopia (compared to no myopia). The reason for a combination of periodontal health and stage I periodontitis into a group was due to the periodontal health and stage I periodontitis status as the goal of the 2017 world workshop for stage II-IV periodontitis treatments. In addition, we separated periodontal health and stage I periodontitis into two groups and compared the risk of myopia with stage II/III periodontitis via a sensitivity test. In Model 1, age, BMI, systolic BP, smoking status and alcohol intake status and missing teeth numbers were adjusted. In Model 2, further adjustments were made, including blood leucocyte counts, triglycerides, high-density lipoprotein and uric acid levels. The covariates for both dental diseases and myopia (including age, metabolic syndrome components, toxic substance consumption and inflammatory markers selected in Models 1 and 2) were based on the findings from previous studies [7, 20–22, 25] and the variables of the three myopic groups for a comparison with a p value < 0.15. Additionally, missing teeth numbers related to the removal of severely dental caries and periodontitis were adjusted in Models 1 and 2. SPSS statistical software (IBM Corp., Version 25.0. International Business Machines Corporation, Armonk, NY, USA) was used for all of the statistical analyses.

## Results

**Fig. 1:**
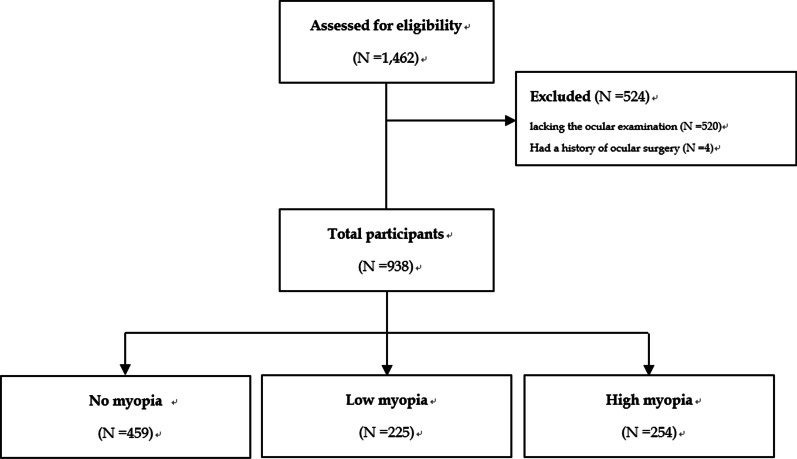
The flow diagram for the inclusion of study participants

In total, there were 1,462 military participants receiving the oral examinations. After excluding those without an ocular examination (N = 520) in the same year and those who had a history of ocular surgery (N = 4), a sample of 938 military male and female subjects aged 19–39 years was included for the final analysis. These individuals were subsequently divided into three groups: no myopia (N = 459), low myopia (N = 225) and high myopia (N = 254), as shown in Fig. 1.

Table 1 shows the characteristics of the participants. In general, there were similar data regarding demographics, anthropometrics and toxic substance use among the three groups except for a higher prevalence of active smoking and related grade B and C periodontitis in participants without myopia. In addition, participants with high myopia had higher BP, blood leukocyte counts and serum uric acid levels than those without myopia or with low myopia. The DMFT index and periodontitis severity were similar among the three myopic groups, except for the fact that the number of filled teeth was significantly lower in those patients without myopia.

**Table 1:** Baseline characteristics of the study cohort (N = 938)

	No myopia (N = 459)	Low myopia (N = 225)	High myopia (N = 254)	*P* value
Male (%)	399 (86.9)	199 (88.4)	218 (85.8)	0.69
Age (years)	30.26 ± 5.72	30.69 ± 5.12	31.21 ± 6.11	0.10
Smoking, active (%)	103 (22.4)	43 (19.1)	36 (14.2)	0.02
Alcohol intake, active (%)	104 (22.7)	56 (24.9)	44 (17.3)	0.10
Body mass index (kg/m^2^)	26.17 ± 3.65	25.72 ± 4.00	25.59 ± 3.81	0.10
Waist circumference, cm	86.05 ± 10.26	84.60 ± 10.64	87.38 ± 11.59	0.01
Systolic blood pressure (mmHg)	122.21 ± 12.85	118.14 ± 12.42	123.67 ± 12.57	< 0.001
Diastolic blood pressure (mmHg)	73.87 ± 10.96	72.26 ± 9.31	74.93 ± 10.33	0.02
Refractive error (diopter) for right eye	0.00 ± 0.05	-2.92 ± 1.59	-6.50 ± 0.42	< 0.001
Refractive error (diopter) for left eye	0.00 ± 0.05	-2.82 ± 1.65	-6.39 ± 0.86	< 0.001
*Blood biochemical tests*				
Leucocyte count (10^3^/mm^3^)	7.16 ± 1.74	6.80 ± 1.58	7.01 ± 1.81	0.03
Serum uric acid (mg/dl)	6.78 ± 1.49	6.36 ± 1.50	6.50 ± 1.33	0.001
Serum triglycerides (mg/dl)	142.49 ± 120.86	124.03 ± 86.69	134.22 ± 111.61	0.12
Total cholesterol (mg/dl)	183.02 ± 34.54	180.04 ± 35.05	184.25 ± 35.95	0.40
Fasting glucose (mg/dl)	93.44 ± 16.71	93.67 ± 13.81	93.13 ± 21.83	0.94
High-density lipoprotein (mg/dl)	48.46 ± 9.90	48.73 ± 11.06	49.92 ± 11.92	0.21
Low-density lipoprotein (mg/dl)	110.74 ± 29.61	108.77 ± 31.49	109.27 ± 31.17	0.68
*DMFT index*				
Actively dental caries numbers	0.48 ± 1.19	0.64 ± 1.37	0.67 ± 1.51	0.13
Missing teeth numbers	0.92 ± 1.57	0.86 ± 1.34	0.80 ± 1.51	0.59
Filled teeth numbers	3.32 ± 3.49	4.14 ± 3.84	3.45 ± 3.35	0.01
Participants with any actively decayed teeth	110 (24.0)	66 (29.3)	68 (26.8)	0.30
*Periodontitis severity*				
Healthy	287 (62.5)	131 (58.2)	149 (58.7)	0.11
Stage I	49 (10.7)	30 (13.3)	19 (7.5)	
Stage II/III	123 (26.8)	64 (28.4)	86 (33.9)	
Periodontitis grade*				
A	356 (77.6)	182 (80.9)	218 (85.8)	0.03
B	77 (16.8)	36 (16.0)	23 (9.1)	
C	26 (5.7)	7 (3.1)	13 (5.1)	
Probing pocket depth, PPD (mm)^‡^	2.35 ± 0.47	2.77 ± 0.66	2.79 ± 0.98	0.19
Sites with PPD 4–5 mm (%)	1.22 ± 1.70	1.16 ± 1.69	1.58 ± 1.32	0.28
Sites with PPD ≥ 6 mm (%)	0.19 ± 0.53	0.29 ± 0.53	0.32 ± 0.76	0.22
Clinical attachment loss, CAL (mm)^‡^	2.55 ± 0.66	3.02 ± 0.54	3.12 ± 0.58	0.22
Sites with CAL 3–4 mm (%)	2.67 ± 0.89	2.66 ± 0.31	2.65 ± 0.79	0.19
Sites with CAL ≥ 5 mm (%)	1.49 ± 1.93	1.74 ± 1.66	1.95 ± 1.56	0.34

Continuous variables are expressed as mean ± standard deviation, and categorical variables as n (%). Analysis of variance was used to compared the variables among three myopic groups

DMFT, numbers of decayed, missing and filled teeth

^*^ For the grade of periodontitis, all participants were classified as grade A, according to merely the primary criteria of radiologic fining for bone loss/age (%) < 0.25. If the grade modifier of smoking status taken into account, the smokers with < 10 cigarettes/day were classified as grade B, and the smokers with ≥ 10 cigarettes/day were classified as grade C

^‡^ The definition for sites with specific levels of PPD (%) and CAL (%) were the numbers of site fit the criteria divided by the total numbers of full mouth sites detected

Table 2 shows the univariate and multivariable linear regression results of refractive error. In the univariate analysis, greater BMI and serum uric acid levels were associated with decreased refractive error (direction to no myopia) (β = 0.05 and β = 0.18, respectively; *p* values = 0.03 and 0.01, respectively). More actively dental caries numbers were marginally associated with increased refractive errors (direction to high myopia) (β = − 0.13, *p* value = 0.07). Missing teeth numbers and filled teeth numbers and probing pocket depth were not associated with refractive errors. Actively dental caries numbers, missing teeth numbers, age, BMI, systolic BP, blood leukocyte counts and serum uric acid levels constructed the multivariable linear regression model. However, filled teeth numbers and probing pocket depth were eliminated due to the threshold *p*-value > 0.30 using backward regression selection. In the multivariable linear regression model, greater actively dental caries numbers, age, systolic BP and lower serum uric acid levels were associated with increased refractive errors (direction to high myopia) (β = − 0.17, β = − 0.04, β = − 0.02 and β = 0.18, respectively; all *p* values < 0.05). In addition, greater missing teeth numbers and BMI levels were marginally associated with lower refractive errors (direction to no myopia) (β = 0.11 and β = 0.06, respectively; *p* values = 0.09 and 0.06, respectively).

**Table 2:** Liner regression analysis models for refractive error

	Univariate				Multivariate		
	R	β	95% CI	*p* value	β	95% CI	*p* value
Actively dental caries numbers	0.06	− 0.13	− 0.27, 0.01	0.07	− 0.17	− 0.31, − 0.03	0.02
Missing teeth numbers	0.03	0.06	− 0.06, 0.18	0.33	0.11	− 0.02, 0.24	0.09
Filled teeth numbers	0.03	− 0.02	− 0.07, 0.03	0.42			
Probing pocket depth	0.01	0.03	− 0.36, 0.43	0.86			
Age	0.05	− 0.03	− 0.06, 0.01	0.12	− 0.04	− 0.08, − 0.01	0.01
Body mass index	0.07	0.05	0.01, 0.10	0.03	0.06	− 0.01, 0.12	0.06
Systolic blood pressure	0.04	− 0.01	− 0.02, 0.01	0.27	− 0.02	− 0.03, − 0.00	0.02
Blood leucocyte counts	0.05	0.08	− 0.03, 0.19	0.16	0.06	− 0.05, 0.18	0.27
Serum triglycerides	0.03	0.01	− 0.01, 0.00	0.33			
High density lipoprotein	0.08	− 0.02	− 0.03, 0.00	0.06			
Serum uric acid	0.09	0.18	0.05, 0.31	0.01	0.18	0.04, 0.32	0.01

Multivariable linear regression model was constructed by backward stepwise selection method

Table 3 displays the results of the multiple logistic regression analysis for the presence of actively dental caries, filled teeth and stage II/III periodontitis with low and high myopia. Although the association of the presence of any actively dental caries with any myopia (low and high) was not significant in the crude model, the association was marginally significant in model 1 (OR and 95% CI 1.33 [0.98–1.81]) and was significant in Model 2 (OR: 1.42 [1.04–1.94]), probably due to the presence of confounders between the myopic groups. In addition, in Model 2, the associations between the presence of any actively dental caries, low myopia and high myopia were similar (OR: 1.43 [0.98–2.10] and 1.39 [0.96–2.02], respectively). By contrast, the presence of stage II/III periodontitis was only associated with high myopia rather than low myopia in Model 1 (OR: 1.47 [1.04–2.08] and 0.97 [0.66–1.41], respectively) and Model 2 (OR: 1.52 [1.07–2.15] and 1.01 [0.69–1.48], respectively). However, there was no association between the presence of filled teeth and any myopia.

**Table 3:** Multivariate logistic regression analysis model for low and high myopia (no myopia as reference) with actively decayed teeth, filled teeth and stage II/III periodontitis

	Any myopia			Low myopia			High myopia		
	OR	95% CI	*p* value	OR	95% CI	*p* value	OR	95% CI	*p* value
*Presence of any actively dental caries*									
Crude	1.22	(0.91, 1.64)	0.17	1.32	(0.92, 1.88)	0.13	1.16	(0.82, 1.65)	0.40
Model 1	1.33	(0.98, 1.81)	0.07	1.33	(0.91, 1.93)	0.14	1.32	(0.92, 1.91)	0.13
Model 2	1.42	(1.04, 1.94)	0.02	1.43	(0.98, 2.09)	0.07	1.39	(0.96, 2.01)	0.08
*Presence of any filled teeth*									
Crude	1.18	(0.88, 1.57)	0.26	1.34	(0.93, 1.94)	0.11	1.06	(0.75, 1.49)	0.73
Model 1	1.06	(0.78, 1.43)	0.70	1.23	(0.83, 1.81)	0.30	0.93	(0.65, 1.34)	0.70
Model 2	1.03	(0.76, 1.40)	0.83	1.17	(0.79, 1.73)	0.44	0.93	(0.65, 1.33)	0.68
*Presence of stage II/III periodontitis*									
Crude	1.17	(0.88, 1.55)	0.28	0.94	(0.65, 1.35)	0.72	1.39	(0.99, 1.94)	0.05
Model 1	1.22	(0.91, 1.64)	0.18	0.97	(0.66, 1.41)	0.85	1.47	(1.04, 2.08)	0.02
Model 2	1.27	(0.94, 1.71)	0.11	1.01	(0.69, 1.48)	0.96	1.52	(1.07, 2.15)	0.01

Data are presented as odds ratios (OR) and 95% CI (confidence intervals) using multiple logistic regression analysis for Model 1: sex, age, BMI, SBP, smoking, drinking and missing teeth, Model 2: sex, age, BMI, SBP, smoking, drinking, missing teeth, leucocyte count, serum triglyceride, serum uric acid and high-density lipoprotein

The Additonal file 1: Table 1 shows the multiple logistic regression analysis results of the sensitivity test for the association of stage I and stage II/III periodontitis (the healthy individuals were used as the reference) with myopia. There was no association of stage I periodontitis with low and high myopia. In contrast, an association between stage II/III periodontitis and high myopia was suggested (OR: 1.41 [0.99–2.01], *p* = 0.054) and consistent with the results found in Table 3.

## Discussion

The main findings of the present study were that actively dental caries were a risk factor for myopia. However, dental caries after complete treatment and without recurrence (filled teeth) were not risk factors for myopia. In addition, there was an association of more severe periodontitis with high myopia. In contrast, an association between more severe periodontitis and low myopia was not observed in young adults.

The association of dental caries with myopia has been studied since the 1970s, and the conclusions have been inconsistent in previous reports. Studies for children and teenagers demonstrated no association of dental caries with myopia [14, 15], which was in contrast to the findings for young adults [12, 13]. This may be partially explained by the fact that the prevalence of myopia is greater in young adults than in young adults [4]. In contrast, the incidence of actively dental caries was higher in youth than in young adults, which is likely due to poor oral hygiene in youth [26]. In addition, the present study revealed that dental caries after complete treatment (filled teeth) were no longer associated with myopia. Active dental caries are caused by poor oral hygiene and bacterial growth in the teeth [27], which can lead to dental destruction and systemic inflammation [11]. If the actively dental caries were treated, the impact of systemic inflammation on lens fibrosis would be much reduced [28], thus decreasing the risk of myopia [29]. Previous studies have also proposed that the submalnutrition status reflected by a lower BMI level may weaken both the teeth enamel and the lens collagen tissue, thereby establishing the association between dental caries and myopia, which was also observed in the present study. The other possible mechanism for the association could also be explained by the present study findings that greater levels of serum uric acid (which is an antioxidant agent in the body) may protect against oxidation stress [30] from both myopia and tooth decay in young adults.

The mechanism of periodontal tissue destruction is highly related to the immune response but not via a direct consequence of the bacteria [20–22]. With the stimulation of oral biofilms, the host’s own inflammatory cells and fibroblasts secrete matrix metalloproteinases (MMPs) and proinflammatory cytokines, such as tumor necrosis factor-α, interleukin (IL)-1, IL-6 and leptin, which result in periodontal tissue destruction [20–22]. Local inflammation can also affect systemic health [31]. MMP-2, which is one target of transforming growth factor (TGF) β signaling via NFκB, can cleave collagens, thereby promoting the progression of myopia [32, 33].

The development of periodontitis is also associated with genetic bases and lifestyles (especially smoking and poor oral hygiene), which allow bacteria to express their pathogenic potential [34, 35]. The study for identical twins who were raised apart from each other revealed that 38%-80% of the population variance for periodontal disease may be explained by genetic factors, and the heritability remained significant with adjustments for behavioral variables, e.g., smoking [36]. Interleukin genes, formyl peptide receptor genes and Fc gamma receptor genes are believed to be associated with periodontal disease [37]. Similar to periodontitis, high myopia is etiologically heterogeneous and results from complicated interactions between genetic and environmental factors [38]. All human and animal studies have strongly suggested that environmental factors play an important role in the development and progression of myopia; however, in human population studies, the contribution of genetic factors accounted for at least 70% of the variance in refraction [39]. High myopia and periodontal disease may share common genetic defects and behavior factors, including inactivity, which can provide a link between the two diseases.

The present study had some limitations. For example, this study was a cross-sectional design, and temporality and causality could not be assessed. Additionally, less time outdoors and being closer to work can increase the risk of myopia [40]; however, the military health examination did not provide this information. Moreover, more basic experiments are required to elucidate the physiological mechanism between myopia, dental caries and periodontitis. Finally, due to the fact that our study subjects were young adults, the generalizability may not be applicable to other age spectra. In contrast, there were several strengths to the present study. First, this was the first report to show an association between a higher severity of periodontitis and high myopia in young adults. In addition, another main contribution of the present study in the dental field was to clarify the gap between previous studies in which both actively decayed and filled teeth were integrated into the “dental caries” for assessing the risk of myopia. Finally, as certain military lifestyle factors, such as diet and physical training, were similar, many unmeasured confounders were adjusted at baseline. Compared to the general population of young adults, the military personnel were more physically fit, and spent less time in reading documents and watching television, possibly reducing the bias which may lead to myopia, and thus reinforcing the link to dental caries.

## Conclusions

In conclusion, our findings suggest that only actively dental caries and stage II/III periodontitis were associated with myopia among young adults, thus highlighting the dental inflammation status in the oral cavity as a potential link to myopia. The treatment of actively dental caries and severe periodontitis in young adults may be vital for the primary prevention of myopia progression.

## Supplementary Information

**Additional file 1: Table 1.** Association of Stage I and Stage II/III Periodontitis With Myopia

## Data Availability

The datasets generated and/or analysed during the current study are not publicly available due to materials obtained from the military in Taiwan, which were confidential, but are available from the corresponding author on reasonable request.
