# Supplementary material for: Dental caries and periodontitis and the risk of myopia in young adults: CHIEF oral health study
Source: BMC Oral Health. 2022 Sep 5;22:384. doi: 10.1186/s12903-022-02413-w (PMC9446871; doi:10.1186/s12903-022-02413-w)
Supplement: Supplementary file 1 — Additional file 1: Table 1. Association of Stage I and Stage II/III Periodontitis With Myopia [file 12903_2022_2413_MOESM1_ESM.docx]

Supplemental Table 1. Association of Stage I and Stage II/III Periodontitis With Myopia

|  | Any myopia | | |  | Low myopia | | |  | High myopia | | |
| --- | --- | --- | --- | --- | --- | --- | --- | --- | --- | --- | --- |
|  | OR | 95% CI | *P*-value |  | OR | 95% CI | *P*-value |  | OR | 95% CI | *P*-value |
| Periodontitis |  |  |  |  |  |  |  |  |  |  |  |
| Model 1 |  |  |  |  |  |  |  |  |  |  |  |
| Healthy | 1.00 |  |  |  | 1.00 |  |  |  | 1.00 |  |  |
| Stage I | 1.09 | 0.70 – 1.68 | 0.71 |  | 1.34 | 0.81 – 2.24 | 0.25 |  | 0.87 | 0.49 – 1.56 | 0.64 |
| Stage II/III | 1.27 | 0.94 – 1.70 | 0.11 |  | 1.12 | 0.76 – 1.63 | 0.56 |  | 1.39 | 0.98 – 1.97 | 0.06 |
| Model 2 |  |  |  |  |  |  |  |  |  |  |  |
| Healthy | 1.00 |  |  |  | 1.00 |  |  |  | 1.00 |  |  |
| Stage I | 1.11 | 0.71 – 1.72 | 0.65 |  | 1.38 | 0.82 – 2.31 | 0.22 |  | 0.90 | 0.50 – 1.61 | 0.71 |
| Stage II/III | 1.28 | 0.95 – 1.72 | 0.10 |  | 1.11 | 0.75 – 1.62 | 0.61 |  | 1.41 | 0.99 – 2.01 | 0.05 |

Data are presented as odds ratios (OR) and 95% CI (confidence intervals) using multiple logistic regression analysis for

Model 1: sex, age, body mass index, systolic blood pressure, smoking status, alcohol drinking status and missing teeth numbers

Model 1: sex, age, body mass index, systolic blood pressure, smoking status, alcohol drinking, status missing teeth numbers, blood leucocyte count, triglycerides and uric acid
